# Sensitivity of Ethiopian aquatic macroinvertebrates to the pesticides endosulfan and diazinon, compared to literature data

**DOI:** 10.1007/s10646-016-1676-0

**Published:** 2016-05-24

**Authors:** Berhan M. Teklu, Negussie Retta, Paul J. Van den Brink

**Affiliations:** Department of Aquatic Ecology and Water Quality Management, Wageningen University, Wageningen University and Research centre, P.O. Box 47, 6700 AA Wageningen, The Netherlands; College of Natural Sciences, University of Addis Ababa, 4 Kiklo Campus, Addis Ababa, Ethiopia; Alterra, Wageningen University and Research centre, P.O. Box 47, 6700 AA Wageningen, The Netherlands

**Keywords:** Single-species toxicity tests, Tropics, Ecological risk assessment, Species sensitivity distribution, Africa

## Abstract

The aims of the present study were to present a methodology for toxicity tests that can be used when analytical resources to verify the test concentrations are limited, and to evaluate whether the sensitivity of a limited number of Ethiopian species to pesticides differs from literature values for, mainly, temperate species. Acute toxicity tests were performed using three Ethiopian aquatic invertebrate species, one crustacean (*Diaphanosoma brachyurum*) and two insects (*Anopheles pharoensis* and *Culex pipiens*) and using the pesticides endosulfan and diazinon. All species–pesticide combinations were tested in duplicate to estimate the consistency, i.e. the intra-laboratory variation, in test results. *Daphnia magna* was tested as well to allow the test results to be compared directly with values from the literature. Results indicate that the differences between the EC50s obtained for *D. magna* in this study and those reported in the literature were less than a factor of 2. This indicates that the methodology used is able to provide credible toxicity values. The results of the duplicated tests showed intra-laboratory variation in EC50 values of up to a factor of 3, with one test showing a difference of a factor of 6 at 48 h. Comparison with available literature results for arthropod species using species sensitivity distributions indicated that the test results obtained in this study fit well in the log-normal distribution of the literature values. We conclude that the methodology of performing multiple tests to check for consistency of test results and performing tests with *D. magna* for comparison with literature values to check for accuracy is able to provide reliable effect threshold levels and that the tested Ethiopian species did not differ in sensitivity from the arthropod species reported on in the literature.

**Electronic supplementary material:**

The online version of this article (doi:10.1007/s10646-016-1676-0) contains supplementary material, which is available to authorized users.

## Introduction

The current intensification of agricultural activities in Ethiopia results in a steady increase in both the types and quantities of agrochemicals (Taddese and Asferachew 2008). Pesticides may, however, cause risks to aquatic ecosystems through contamination by spray drift, run-off, drainage and accidental spills. To prevent environmental harm from the application of these agrochemicals, it is essential to perform a prospective environmental risk assessment before registering a pesticide (Teklu et al. 2015). Estimating the risks of pesticides to the aquatic ecosystem includes an effect assessment which is often based on acute and chronic laboratory tests of the toxicity of these compounds to aquatic species. Brock et al. (2006) noted the importance of acute toxicity tests with fish, algae and invertebrates for the first tier in the risk assessment of pesticides, in order to identify ecosystem components whose sensitivity should be further evaluated in higher-tier risk assessment procedures (Van den Brink 2013). These tests also help the retrospective chemical risk assessment, by identifying species that are sensitive to pesticide pollution, so that the presence or absence of a sensitive species in an area may be an indication of the pollution status of that particular area (e.g. Wahizatul et al. 2011), although the absence of a species may have other causes as well.

At present, such an assessment often depends on the results of toxicity tests performed with temperate species, as data on tropical species are scarce (Kwok et al. 2007). Risk assessments performed for tropical ecosystems should be (partially) based on toxicity data for tropical species, since differences in sensitivity might be expected (Daam and Van den Brink 2010), although empirical data suggest no systematic differences in sensitivity (e.g. Kwok et al. 2007; Rico et al. 2010). Gathering sensitivity data for local species enables further examination of whether European and North American data can be extrapolated to other geographical areas (Hose and Van den Brink 2004; Maltby et al. 2005). Although Ethiopia is located in the tropical region, the risk assessment for pesticide registration is solely dependent on the available temperate acute toxicity data (Teklu et al. 2015). Only a few toxicity tests have been performed with Ethiopian species, one example being a study evaluating the effect of the poisonous extract of the plant *Millettia ferruginea* on Baetidae (mayflies) and Hydropsychidae (caddisflies) (Karunamoorthi et al. 2009).

Besides the laboratory infrastructure needed to perform these tests, one other challenge to conducting such tests in a developing country like Ethiopia is the availability of analytical equipment and the costs of analyses to verify the test concentrations used in the experiments. In this paper we present a simple methodology that circumvents the need for test concentration verification, which might be helpful for future aquatic risk assessment in Ethiopia or elsewhere in the developing world, where the availability of analytical laboratory equipment is limited. The proposed methodology includes performing multiple tests to check for consistency of test results and performing tests with *Daphnia magna* for comparison with literature values to check for accuracy.

The objectives of the current study were (i) to produce toxicity data for local Ethiopian species, (ii) to compare the sensitivity of the Ethiopian species with literature data which relates mainly to temperate species and (iii) to present a simple methodology for conducting tests which reduces the need for analytical verification of the exposure concentrations.

## Materials and methods

### Test compounds

One organochlorine (endosulfan) and one organophosphate (diazinon) insecticide were chosen as model compounds to evaluate the effects of pesticides on Ethiopian aquatic macroinvertebrates. This choice was based on their frequency of use in Ethiopia, available temperate toxicity data and the results of a previously performed risk assessment for Ethiopian aquatic ecosystems (Teklu et al. 2015). The pesticides, containing 99 % active ingredient (endosulfan or diazinon) were obtained from the Adami Tulu Pesticide Processing S.C. in Addis Ababa, Ethiopia.

### Test organisms

The sensitivity of two crustaceans (*D. magna* and *Diaphanosoma brachyurum)* and two insect species (*Anopheles pharoensis* and *Culex pipiens*) was assessed for both endosulfan and diazinon. *D. magna* individuals were obtained from the National Fisheries and Aquaculture Research Centre (part of the Ethiopian Institute of Agricultural Research) while *D. brachyurum* and *A. pharoensis* were collected from the Koka area and *C. pipiens* from the Entoto natural park located in the periphery of Addis Ababa. Insect larvae were kept for 2 days for acclimatization in the laboratory in a tray with water from the collection site and introduced to test water. Second and third instar larvae were used in the tests. All collected larvae were maintained in the laboratory until emergence, and the flying adult stage was used for further identification. *D. magna* and *D. brachyurum* were cultured in the laboratory in a culturing dish with water from the collection site, and the tests were started when enough individuals of similar size and age category were available. All arthropods were identified using a standard identification key and in consultation with Addis Ababa University experts (Hopkins 1952; Verrone 1962). *D. magna* was selected because it is the most important international standard test species and could thus be used to validate the test performance against literature data. The other species were selected based on their availability and non-cannibalistic behaviour and to include species from both the insect and the crustacean groups. Second instar individuals were used for the tests with the insect species, while individuals younger than 24 h were used for the tests with the crustaceans.

### Toxicity tests

All toxicity experiments were performed at the Fisheries and Limnology Laboratory of Addis Ababa University College of Natural Sciences, in accordance with the OECD’s *Daphnia* sp. protocol (OECD 2004). The acute toxicity tests were performed using seven concentrations (including control), with three replications per treatment. The toxicity tests used a static exposure extended for 96 h with a single pesticide spiking at the beginning of the test while establishing treatments. The dissipation from the water phase during the 96 h experimental test period was expected to be low for endosulfan (DT50_hydrolysis_ = 20 days, pH 7, T = 20 °C, Lewis et al. 2016) and 50 % for diazinon (DT50_hydrolysis_ = 138 days, pH 7, T = 20 °C, Lewis et al. 2016). All tests were done in 1.5 L glass jars filled with 1 or 0.5 L of water for insects and crustaceans, respectively. The crustaceans used were the cultured individuals, while insects were introduced into the test water after two days of acclimatization. Dechlorinated tap water was used for the crustaceans, while insects were tested in filtrated (mesh size 1–1.5 nm) water from the collection site. Stock solutions of 100 mg/L were prepared using demineralised water for both pesticides, using absolute ethanol (0.1 %) as a solvent in view of the low and moderate water solubility of the substances (Lewis et al. 2016), leading to an ethanol content of 0.003 % at the highest endosulfan concentration and of 0.0004 % at the highest diazinon concentration. Test concentrations were prepared following successive serial dilutions stirred thoroughly for 15 s for each replication. Concentrations were chosen in such a way that no effects were expected at the lowest concentration and 100 % effects at the maximum concentration, using a published EC50 value from a related temperate species as a reference, while the concentrations in between were geometrically spaced (see Supplementary Information for the concentrations evaluated). In each test, ten individuals were added to each replicate, assuming non cannibalistic behaviour of all test species. During the experiments the average temperature was 20 ± 0.25 °C, dissolved oxygen (DO) averaged 4.57 ± 0.41 mg/L and pH 6.52 ± 0.13. Measurements were performed at the beginning and end of the experiment using a portable DO (Handy Polaris, OxyGuard, USA) and pH meter (WTW multi 340i, USA).

Invertebrate immobility was taken as an endpoint for assessing the effects of endosulfan and diazinon, as described in Rubach et al. (2011). All pesticide–species combinations were tested twice, except for the test with endosulfan and *D. magna*. In most tests, counting was done every 24 h until the end of the test (96 h), while two tests were only evaluated after 48 and 96 h (see Supplementary Material).

### Analytical verification of stock solution

Samples of the stock solutions were taken to Wageningen (The Netherlands) in glass vials for analytical verification of the concentration. Some decrease in the concentration of the stock solutions of diazinon and endosulfan was expected as they were stored for 9 months in a fridge at 5 °C at an Addis Ababa University laboratory and for 3 months frozen (in Wageningen). To verify the actual concentration of the stock solutions (100 mg/L) at the time of testing, the expected degradation during the storage period was calculated using the degradation rates available in the literature. It was assumed that no degradation took place while frozen, since both pesticides are moderately volatile given their saturated vapour pressure and associated Henry coefficients of 0.000025 (diazinon) and 0.00043 (endosulfan) (Lewis et al. 2016). The DT50 values of endosulfan and diazinon found in the literature for a pH of 5 were established at 25 and 20 °C, respectively. The effect of the lower temperature in the fridge (5 °C) on the degradation was accounted for using a correction to the rate determined at reference conditions. This was calculated with the Arrhenius equation (Boesten 1986), using the molar Arrhenius activation energy for hydrolysis of pesticides in water, 75,000 J/mol (Deneer et al. 2010). Since the degradation of diazinon is expected to be mainly driven by hydrolysis and, therefore, pH dependent, we also measured the pH of the solution before analytical verification of the test compound.

After storage of the stock solutions in Addis Ababa and Wageningen, the concentrations in the stock solutions were verified by means of GC–ECD. For this purpose, dilutions of the stock solutions were prepared for both diazinon (50-fold) and endosulfan (10,000-fold). Samples (3 µL) were injected at an inlet temperature of 250 °C with a split ratio of 1:20 on a HP5MS column (15 m × 0.25 mm with 0.25 µm film thickness). The oven was operated under isothermal conditions at a temperature of 180 °C, while the ECD was set at a temperature of 300 °C. The retention times were found to be 8.15 min. (diazinon) and 12.35 min. (endosulfan), respectively. Calculation of the concentrations was based on external standards.

### Data analysis

The EC10 and EC50 values and their 95 % confidence intervals were estimated after 24, 48, 72, and 96 h by log-logistic regression using the number of immobile individuals per replicate as input (Rubach et al. 2011). The test was considered valid when the immobilisation observed in the controls was 10 % or less at 48 h and 20 % or less at 96 h. The 48 h value is based on the acceptance criteria of the OECD protocols, which is 10 % for the 24 h *Daphnia* sp. test and 15 % for the 48 h *Chironomus* sp. test. The test results were considered to be invalid when a control immobilisation higher than 10 % was observed at 48 h or higher than 20 % was observed at 96 h. Results from duplicate tests were considered to be different when the 96 h EC50 values differed by a factor of 3 or more (Boxall et al. 2001; Baird et al. 1989). Since some of the 96 h test results were invalid, the comparisons were also made based on 48 h values. The 48 and 96 h EC50 values of *D. magna* were compared in the same way with those reported in the ECOTOX data base (www.epa.gov/ecotox, assessed on 22 Dec 2015).

In order to compare our findings with those available in the literature, species sensitivity distributions (SSDs) were constructed for each combination of exposure time (48 and 96 h) and pesticide (endosulfan and diazinon) (Posthuma et al. 2002). This was done using the ETX2.0 program (Van Vlaardingen et al. 2004), which fits a log-normal model to the data. For each SSD, the median 50 and 5 % hazardous concentration (HC50 and HC5) and its standard deviation were calculated. The goodness-of-fit was tested using the Anderson–Darling test for normality. The data used for the construction of the SSDs of both pesticides for arthropods were extracted from the valid test results from the current study and the ECOTOX database (www.epa.gov/ecotox, accessed on 22 Dec 2015). When multiple values were available for the same species, the geometric mean was calculated.

## Results and discussion

### Analytical verification of stock solution

According to the analytical results, diazinon had decreased from the nominal concentration of the stock solution of 100 mg/L to a concentration as low as 0.70 mg/L at pH 4, while endosulfan had decreased to 80 mg/L at pH 5 during the 9 months stay in the fridge at 5 °C (Table 1).

**Table 1:** DT50 values (days) and calculated and measured concentrations (mg/L) of the pesticide stock solutions of 100 mg/L after 9 months of storage in the freezer, and pH of the measured samples

	Endosulfan	Diazinon
Literature DT50 at pH 5	91 (T = 25 °C) (Fan 2008)	12 (T = 20 °C) (Lewis et al. 2016)
Calculated DT50 at pH 5 and 5 °C	806	62
Calculated concentration after 9 months at 5 °C	79	4.6
Measured concentration after 9 months at 5 °C	80	0.70
pH of the sample	5	4

The literature provided DT50 values for both chemicals for a pH of 5 (Table 1). After correction for the temperature, endosulfan was predicted to be very stable at 5 °C (DT50 = 806 days), while the half-life of diazinon was much shorter (62 days). The calculated concentrations for both chemicals were in the same range as the measured ones (Table 1). The mismatch between the calculated concentration for diazinon of 4.6 μg/L and the measured concentration of 0.70 μg/L might be explained by the lower pH in the sample (4) than for which the DT50 was determined (5), since it may be expected that the hydrolysis rate of diazinon is higher at a pH of 4 than of 5 (Lewis et al. 2016). This means that it is likely that at the start of the experiments the stock solution was indeed around 100 mg/L for both compounds. We therefore recommend to analytically verify the test concentrations, if this is possible at the test facility, but when this is not possible to at least store some of the stock solution for analytical verification later in a suitable laboratory. It is important to take the properties of the pesticide (e.g. DT50) into account before assuming the validity of this methodology.

### Toxicity of endosulfan and diazinon

For diazinon, the first test with *A. pharoensis* (37 %), *D. brachyurum* (33 %) and *D. magna* (23 %) exceeded the 20 % threshold level at 96 h, while none exceeded the 10 % threshold at 48 h (Table 2; Supplementary Material). All other tests performed showed a control immobilisation <20 % at 96 h, while all performed tests showed control immobilisations of <10 % at 48 h, which is the duration of the test on which the criterion of 10 % is based (OECD 2004). The tests showed large differences between 48 and 96 h values. On average the difference was a factor of 13, while the minimum and maximum factor was 1.8 and 65, respectively. This indicates that, when field exposure is expected to be longer than 2 days, the 48 h toxicity values might not be a good predictor for effects and tests with a longer duration are needed.

**Table 2:** Summary of the results (mean and 95 % confidence interval of EC10 and EC50 in μg/L) of the acute toxicity tests performed with endosulfan and diazinon

Chemical/test organism	Test no.	48 h test results					96 h test results				
		EC10	(conf. int.)	EC50	(conf. int.)	% immo-bilisation	EC10	(conf. int.)	EC50	(conf. int.)	% immo-bilisation
Endosulfan											
*C. pipiens*	1	22.2	(11.5–43.0)	132	(91–190)	0	3.02	(1.16–7.84)	20.1	(12.8–31.4)	0
	2	20.1	(10.1–39.7)	131	(90–192)	0	2.39	(0.788–7.25)	17.0	(10.1–28.8)	3
*A. pharoensis*	1	6.3	(1.96–20.3)	90.7	(52.9–155)	0	5.11	(1.6–16.3)	49.3	(28.7–84.6)	3
	2	8.95	(3.39–23.7)	64.5	(39.4–105)	0	5.00	(1.65–15.2)	25.3	(14.5–44.1)	10
*D. brachyurum*	1	27.1	(XX–XX)	261	(XX–XX)	0	0.178	(0.009–3.55)	10.8	(3.43–34.1)	7
	2	0.346	(0.055–2.20)	203	(52.1–788)	7	0.013	(0–0.528)	17.6	(4.55–68.3)	17
*D. magna*	1	16.8	(4.53–62.6)	181	(98.7–332)	10	10.5	(2.94–37.4)	98.4	(53.1–182)	13
	Lit			356					54		
Diazinon											
*C. pipiens*	1	0.475	(0.101–2.23)	10.6	(5.24–21.4)	7	0.068	(0.009–0.519)	2.38	(1.03–5.51)	7
	2	0.707	(0.22–2.28)	30.1	(11.0–82.4)	0	0.029	(0.005–0.191)	0.943	(0.41–2.17)	10
*A. pharoensis*	1	1.25	(0.404–3.84)	9.25	(5.35–16.0)	7	0.446*	(0.093–2.15)	2.87*	(1.25–6.57)	37
	2	0.146	(0.016–1.35)	19.0	(6.00–60.2)	10	0.037	(0.004–0.376)	3.00	(1.21–7.45)	13
*D. brachyurum*	1	0.126	(0.034–0.476)	1.53	(0.83–2.84)	7	0.041*	(0.006–0.26)	0.316*	(0.122–0.819)	33
	2	0.071	(0.002–3.39)	8.92	(0.865–92.1)	3	0.001	(0–0.02)	0.138	(0.044–0.432)	10
*D. magna*	1	0.037	(0.009–0.154)	0.875	(0.472–1.62)	3	0.003*	(0–0.06)	0.072*	(0.018–0.294)	23
	2	0.007	(0.001–0.07)	1.11	(0.383–3.22)	3	0.001	(0–0.013)	0.089	(0.034–0.236)	10
	Lit			1.3							

The results at 24 and 72 h can be found in the supplementary information together with the estimates of the EC90s and the parameters of the log-logistic model. The geometric mean of the EC50 values for *D. magna* found in the ECOTOX data base are also shown

* Denote that test results are invalid since the control immobilisation is higher than 20 %

The test results for endosulfan indicated that, based on the 96 h values, *C. pipiens* and *D. brachyurum* proved to be the most sensitive species, followed by *A. pharoensis*, while *D. magna* was the least sensitive species (Table 2). In contrast, *D. magna* proved to be the most sensitive organism for diazinon, followed by the other crustacean species and the two insect species (Table 2).

In order to check the accuracy of our test approach we compared the values obtained for *D. magna* with literature values. The ECOTOX database yielded geometric mean 48 and 96 h endosulfan EC50 values of 356 (*n* = 20) and 54 (*n* = 2) µg/L for *D. magna*, respectively. These values are within a factor of 2 of the corresponding values found by us, which were 181 and 98.4 µg/L, respectively (Tables 2, 3). For diazinon, only a 48 h EC50 of 1.30 µg/L for *D. magna* could be calculated from the available data in the ECOTOX database. One test result was deleted from the obtained data, since it resulted in an extremely deviating 48 h LC50, which was 162 times higher than the geometric mean of the other values (*n* = 5). The literature value was within a factor of 2 of the 48 h EC50 values obtained in this study (0.875 and 1.11 µg/L) (Tables 2, 3). Since the variation in threshold levels between laboratories can be substantial due to genetic and environmental variability and their interaction (Baird et al. 1989), the small differences observed here indicate that the lack of analytical verification of the test concentrations did not disqualify the data we found in this study.

**Table 3:** Ratios between the EC50s of the tests performed with the same species and chemicals

Chemical/test organism	Test #	Ratio 48 h EC50	Ratio 96 h EC50
Endosulfan			
*C. pipiens*	1 and 2	1.0	1.2
*A. pharoensis*	1 and 2	1.4	2.0
*D. brachyurum*	1 and 2	1.3	1.6
*D. magna*	Lit and 1	2.0	1.8
Diazinon			
*C. pipiens*	Lit and 1	1.6	
	1 and 2	2.9	2.5
*A. pharoensis*	1 and 2	2.0	NV
*D. brachyurum*	1 and 2	5.9*	NV
*D. magna*	Lit and 1	1.5	NA/NV
	Lit and 2	1.2	NA
	1 and 2	1.3	NV

The values for *D. magna* are compared with a value found in the literature for the same species

*NA* literature data not available, *NV* one of the tests was not valid (see Table 2)

* Ratios higher than 3

In order to check the consistency of our test approach we compared the values obtained in the different tests we performed with the same pesticide–species combinations. Based on the EC50s, only one pesticide–species combination showed a difference larger than a factor of 3 between the test results. The 48 h EC50 values resulting from the two tests performed with *D. brachyurum* and diazinon showed a difference of almost a factor of 6, while no comparison could be made at 96 h because the control immobilisation at 96 h in one of the tests was too high (Tables 2, 3). None of the other tests showed any systematic differences in sensitivity between the 48 and 96 h observations, indicating that the experimental set-up we used resulted in intra-laboratory variations in test results up to a factor of 2 (Table 3), which has also been observed for other laboratories (Boxall et al. 2001).

### Comparison of experimental results with literature data

**Fig. 1:**
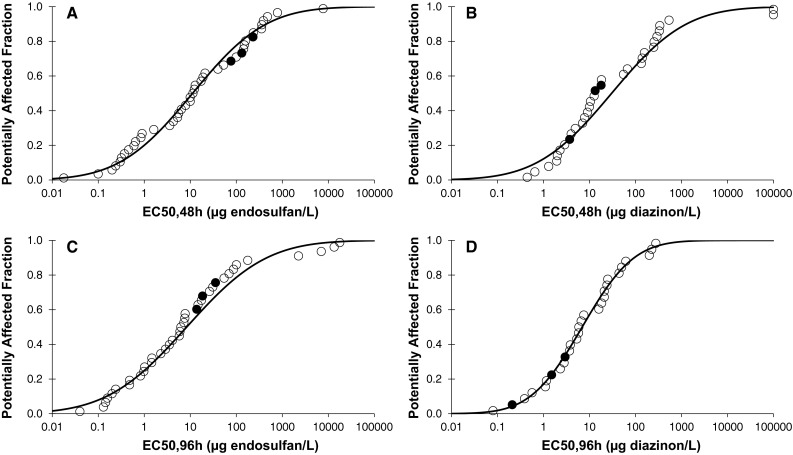
SSD curves for endosulfan (**a**, **c**) and diazinon (**b**, **d**) for 48 h (**a**, **b**) and 96 h (**c**, **d**) toxicity values for arthropods. The *filled symbols* represent the values found in this study. All curves, except **b**, passed all tests of normality at the 0.05 significance level (Anderson–Darling test)

Figure 1 shows the SSDs for endosulfan and diazinon we found for the different observation periods (see Supplementary Information for the included data). Only the 48 h diazinon SSD failed the Anderson–Darling test for normality due to the inclusion of two very insensitive crab species in the SSD. Figure 1 shows that the species tested in this paper were located in the upper (insensitive) part of the endosulfan SSDs, while the same species were located in the middle to lower (sensitive) parts of the diazinon SSDs (Fig. 1). This shows that the toxicity values found in this study are not substantially different from those found in the ECOTOX database and therefore that, at least for these compounds, available data from the literature can be used for an Ethiopian risk assessment, as was done in Teklu et al. (2015).

The endosulfan 48 h HC5 of 0.094 μg/L (0.026–0.26) and the 96 h HC5 of 0.047 μg/L (0.011–0.15) seem lower than the value reported by Hose and Van den Brink (2004) of 0.19 μg/L (0.10–0.59). But this is a result of the use of a different distribution (reciprocal Weibull) since a similar HC5 of 0.083 μg/L (0.017–0.27) is calculated when the same data are fitted by a log-normal distribution, and also pass the Anderson–Darling test. Maltby et al. (2005) reported an HC5 for diazinon of 0.36 μg/L (0.13–0.77), which is only slightly higher than the 48 h HC5 of 0.24 μg/L (0.049–0.76) and the 96 h HC5 of 0.24 μg/L (0.074–0.55) found in our study. This is of course not remarkable, as these values are partly based on the same data.

At the species level, the endosulfan 48 h EC50 value of *D. brachyurum* was in between the other 48 h LC50 values reported for other water fleas, while its 96 h value was a factor of 4 lower than the 96 h LC50 reported for *D. magna* (see Supplementary Information), possibly as a result of the use of another endpoint (immobilisation vs. mortality). The endosulfan 48 h EC50 values reported for the two tested dipteran species (*A. pharoensis* and *C. pipiens*) were quite similar to the 48 h LC50 reported for two other dipterans (*Chironomus riparius* and *Culex fatigans*). No 96 h EC50 or LC50 data were available for other dipteran species, but our results were in between values for other insect (ephemeropteran, plecoptera and zygopteran) species. The 48 h EC50 value of D. brachyurum for diazinon was higher than the 48 h LC50 value reported for other cladoceran species while its 96 h EC50 value was in between the 96 h LC50 values of two other cladoceran species (see Supplementary Information). The 48 and 96 h EC50 values for the two tested dipteran species (*A. pharoensis* and *C. pipiens*) were in between the 48 and 96 h LC50 reported for two other dipterans (*C. riparius* and *Chironomus tentans*). So also at the species level there were no systematic differences in sensitivity present between the species tested in this paper and those found in the ECOTOX database.

### Evaluation of methodology and outlook

The results in the present study provide a methodology for performing single-species acute toxicity studies in developing countries with limited resources to verify the concentrations analytically. By performing a test with *D. magna*, the most tested aquatic species in the world, we enabled the results to be calibrated against literature data. Performing duplicate experiments yields the intra-laboratory variation in test results, which includes the errors made in the dosing of the test systems.

The fact that no systematic differences in test results were found between the Ethiopian species and the values obtained from the literature, which mainly relate to temperate species, does not mean that no test protocols should be developed for indigenous species. Since it is important to test indigenous species, e.g. because they are charismatic, economically important or can be used also for in situ testing, technical protocols should be developed showing how to handle and test indigenous aquatic invertebrates in developing countries.

## Electronic supplementary material

Below is the link to the electronic supplementary material.

Supplementary material 1 (XLSX 62 kb)
